# It is mandatory to review environmental radiofrequency electromagnetic field measurement protocols and exposure regulations: An opinion article

**DOI:** 10.3389/fpubh.2022.992645

**Published:** 2022-10-24

**Authors:** Isabel López, Marco Rivera, Nazario Félix, Ceferino Maestú

**Affiliations:** ^1^Departamento de Fotónica y Bioingeniería (TFB), Escuela Técnica Superior de Ingenieros de Telecomunicación, Universidad Politécnica de Madrid, Madrid, Spain; ^2^Laboratorio de Bioelectromagnetismo, Centro de Tecnología Biomédica, Universidad Politécnica de Madrid, Madrid, Spain; ^3^Departamento de Arquitectura y Tecnología de Sistemas Informáticos (DATSI), Escuela Técnica Superior de Ingenieros Informáticos, Universidad Politécnica de Madrid, Madrid, Spain; ^4^CIBER–BBN Centro de Investigación Biomédica en Red, Madrid, Spain

**Keywords:** non-thermal effects, radiofrequency measurements, frequency, exposimeter, power density

## Introduction

In recent years, there has been a considerable increase in the use of telecommunication technologies. This has led to an increase in the number of users and, consequently, in the number of operational terminals. Mobile networks are continuously improving, which is why, from 2004 to the present, three consecutive generations of mobile networks have been developed (3G, 4G, and 5G) with the consequent improvement in their characteristics, such as higher transmission speed and bandwidths for the transmission of signals. According to the International Telecommunication Union (ITU) World Telecommunication/ICT Indicators Database in 2004 there, were 1.76 billion mobile phone subscriptions in the world, in 2020 this figure increased to 8.27 billion (1). By 2023, there are expected to be 13.1 billion mobile terminals, almost 10% of which will be 5G technology (2).

As technology grows, so does the concern of the public, who feels that they are not adequately informed about the possible effects of long-term exposure to radiofrequency electromagnetic waves (3). There are different sources of radiofrequency electromagnetic field radiation like base stations that allow communication between wireless devices. These base stations are located outdoors on the rooftops of buildings, and indoors like routers, laptops, and mobile phones, using wireless communication technologies such as Bluetooth or WiFi.

Several scientific studies evaluate the possible effects of prolonged exposure to microwaves at the epidemiological level (4–11) and *in vitro* or *in vivo* models (12–19). However, the use of different methodologies in radiation measurement processes and different configurations of exposure equipment, such as frequency, radiation power density, and exposure time; do not allow adequate comparison of results, which makes it difficult to draw conclusions.

The International Commission of Non-Ionizing Radiation Protection (ICNIRP), which is a nongovernmental organization, but recognized by the World Health Organization (WHO), develops international guidelines on exposure limits to EMFs in the range of 0–300 GHz (20). Although most countries such as Spain, Germany, France or Finland adopt the limits proposed by the ICNIRP, the existing regulations in other are different, with limits more restrictive in countries such as Denmark, Bulgaria, Italy, Switzerland, China or Russia (21–23).

## Old standards and measurement methodologies are not adapted to the new communications standards and technologies

There are regulatory organizations in different countries that establish the regulations regarding exposure to EMFs and also offer measurement protocols that attempt to homogenize the sampling process. Some examples are the ICNIRP (20), the ITU (24), the Institute of Electrical and Electronics Engineers (IEEE) (25), the Federal Communication Commission (FCC) (26), or the European Committee for Electrotechnical Standardization (CENELEC) (27).

Most studies related to environmental EMFs in specific populations can be divided into two groups. On the one hand, epidemiological studies assess health parameters such as the presence of headaches, dizziness, sleep problems or others pathologies as a cause of continuous exposure to wireless devices (4–10, 28–35). This first group does not usually perform real-time, *in situ* measurements, and their exposure data is based on the use of different wireless devices, making simple estimates concerning time (28, 31), distance to antennas (29), or mathematical prediction models (36). Secondly, studies that measure in real-time use different protocols and measurement systems (4, 6, 7, 30, 34). On the other hand, publications characterizing EMFs aim to observe that the RF-EMF exposures to which the general population is exposed are within the legal limits of each area (37–40). The main drawback of both kinds of studies is that there is no consensus on the methodology used and, therefore, the results are not comparable or conclusive. Studies of this type require continuous monitoring over time of the evolution of pathologies in the population, as well as any changes that may occur in the radiation levels of the emitting sources.

The characterization of environmental EMFs in a specific population is mostly performed with exposimeter systems such as: EME Spy-−120, 121, 140 (SATIMO, Courtaboeuf, France), ESM 140 (Maschek Electronik, Bad Wörishfen, Germany) or ExpoM—RF (Fields at Work GmbH, Zürich, Switzerland) (41–43). These devices only measure up to 20 spectrum bands, and they do not record the whole frequency spectrum. Furthermore, their bandwidth is determined by the entire frequency band to be measured (41, 44). This does not allow the correct detection of multiple sources that occupy the same communications band, which generally results in a poor estimation of the EMF strength. According to the recommendations of ICNIRP (20) and ITU (45–47), the effect of multiple sources operating at different frequencies should be considered independently, so it is recommended to use systems that allow differentiation of emitting sources using resolution bandwidths determined by the channel width. They also recommend the use of max-hold trace (45) to store the peak value of the measurements made in environmental EMFs, because the RF signals have an irregular or random behavior. Therefore, the use of extrapolation factors in broadband is not enough to compensate the overestimation of the power density of these signals. According to the ITU guidelines for measuring RF-EMF intensity, the average of the signals over 6-min periods should be used to assess compliance of the source(s) (47). However, since the signals behave randomly, so, the information of the maximum, minimum, and average over 6-min periods using wide bandwidths is not sufficient to characterize the EMF strength for different sources. For all of the above reasons, the use of systems such as spectrum analyzers (45) or new exposimeter systems (43) that integrate functionalities of the previous ones that allow discrimination between multiple sources operating at different frequencies, can characterize the behavior of EMFs, obtaining maximum power density per unit of time. These systems would allow the three-dimensional representation of the main RF-EMF characterization parameters: frequency, power density and time.

## Underestimation of non-thermal effects in the development of measurement methodologies

Today, the population is chronically exposed to RF-EMFs, characterized by low intensity, variety, and complexity of signals and long-term exposure durations. ICNIRP sets exposure limits according to the specific absorption rate (SAR) and power density (20). SAR is defined as the rate of energy absorption per defined unit of volume or mass. Many researchers consider the choice of the SAR criterion as the only parameter for assessing effects on biological systems as insufficient, as it only takes into account the thermal effects resulting from exposure (48–50). The ICNIRP guidelines state that a one Celsius degree increase in body temperature is the acceptable limit to avoid adverse health effects (20). Although a reduction factor of 50 is applied to the SAR value capable of increasing the temperature by 1°C, no account is taken of other effects not related to temperature increase. These effects, have been reported in numerous *in vitro* and *in vivo* studies to occur at lower intensities than those required to cause thermal effects by low and high frequency EMFs, such as alterations in gene expression (51, 52), oxidation processes (12, 14, 19, 53–56), the flow of calcium ions (57–59), proteins (60) or cell viability (13, 17).

The first studies to evaluate non-thermal effects were carried out by research groups in the USSR and USA in the 1960s (61–63) and by researchers such as Blackman, Adey, and Bawin later (64–66). An important aspect pointed out by the authors is the modulation parameter that could be responsible for the occurrence of the main effects found in biological systems *in vitro* and *in vivo* as it has a bioactive characteristic and can interfere with some normal and non-linear biological processes. In 1986 the NCRP in its RF exposure guidelines document included a risk assessment with an exception for modulated RF–EMFs (67). However, all other standards, such as those issued by the ICNIRP and the IEEE, ignore the NCRP and revert to considering only the conditions of analysis of thermal effects. The existence of non-thermal effects is reported in those studies using RF-EMF with the same intensity and frequency but with different modulation that find different results with the same experimental setup (68–70). Therefore, existing regulations do not considered a chronic exposure to a pulsed or modulated signal, such as mobile phone signals.

Frequency, defined as the inverse of the wavelength, is an indispensable parameter in the biological response of the cellular body. Research subsequent to that carried out between the 1960s and the 1980s (71–74) allowed us to discuss the existence of frequency and power density windows in which there were biological effects that disappeared with different values of the same parameter, even lower than those proposed by the standards (16, 19, 55, 68, 69).

The studies assessing the possible effects of RF-EMFs *in vitro* should consider all important parameters in the exposure: intensity, frequency, modulation and time of exposure. The possible existence of bioactive windows in frequency, intensity or modulation, as well as the non-linear response of biological systems that could produce differences in cellular behavior should be considered in the development of measurement methodologies and the establishment of exposure limits and could serve as a precedent to establish mechanisms of action of these RF-EMFs in relation to biological systems.

## Conclusions

In conclusion, two main ideas arise: the review of the environmental EMFs measurement protocols and the need for a comprehensive assessment of all the effects of EMFs not only thermal effects.

Measurement protocols must identify the specific frequencies of each of the currently established major frequency bands, the temporal behavior of the signal and the power density. Measurement systems must not only determine averaged field strengths but must be able to measure the peak amplitude over time and, consequently, the cumulative radiation. This would make the characterization of EMFs much more realistic.

In addition, parameters such as frequency and modulation could be important when considering potential biological effects. Choosing intensity as the only determining parameter for the occurrence of effects is a reductionist conception. The consideration of all EMFs parameters in the assessment of biological response should be mandatory.

## Author contributions

IL and MR: drafting the manuscript. MR and NF: revising the manuscript. CM: manuscript direction and supervision. IL, MR, NF, and CM: opinions included in the manuscript. All authors contributed to the article and approved the submitted version.

## Conflict of interest

The authors declare that the research was conducted in the absence of any commercial or financial relationships that could be construed as a potential conflict of interest.

## Publisher's note

All claims expressed in this article are solely those of the authors and do not necessarily represent those of their affiliated organizations, or those of the publisher, the editors and the reviewers. Any product that may be evaluated in this article, or claim that may be made by its manufacturer, is not guaranteed or endorsed by the publisher.
